# Textural properties of microglial activation in Alzheimer’s disease as measured by (R)-[^11^C]PK11195 PET

**DOI:** 10.1093/braincomms/fcad148

**Published:** 2023-05-06

**Authors:** Marta Lapo Pais, Lília Jorge, Ricardo Martins, Nádia Canário, Ana Carolina Xavier, Rui Bernardes, Antero Abrunhosa, Isabel Santana, Miguel Castelo-Branco

**Affiliations:** Coimbra Institute for Biomedical Imaging and Translational Research (CIBIT), Institute for Nuclear Sciences Applied to Health (ICNAS), University of Coimbra, 3000-548 Coimbra, Portugal; Coimbra Institute for Biomedical Imaging and Translational Research (CIBIT), Institute for Nuclear Sciences Applied to Health (ICNAS), University of Coimbra, 3000-548 Coimbra, Portugal; Coimbra Institute for Biomedical Imaging and Translational Research (CIBIT), Institute for Nuclear Sciences Applied to Health (ICNAS), University of Coimbra, 3000-548 Coimbra, Portugal; Coimbra Institute for Biomedical Imaging and Translational Research (CIBIT), Institute for Nuclear Sciences Applied to Health (ICNAS), University of Coimbra, 3000-548 Coimbra, Portugal; Clinical Academic Centre of Coimbra (CACC), Faculty of Medicine (FMUC), University of Coimbra, 3000-548 Coimbra, Portugal; Coimbra Institute for Biomedical Imaging and Translational Research (CIBIT), Institute for Nuclear Sciences Applied to Health (ICNAS), University of Coimbra, 3000-548 Coimbra, Portugal; Coimbra Institute for Biomedical Imaging and Translational Research (CIBIT), Institute for Nuclear Sciences Applied to Health (ICNAS), University of Coimbra, 3000-548 Coimbra, Portugal; Clinical Academic Centre of Coimbra (CACC), Faculty of Medicine (FMUC), University of Coimbra, 3000-548 Coimbra, Portugal; Coimbra Institute for Biomedical Imaging and Translational Research (CIBIT), Institute for Nuclear Sciences Applied to Health (ICNAS), University of Coimbra, 3000-548 Coimbra, Portugal; Clinical Academic Centre of Coimbra (CACC), Faculty of Medicine (FMUC), University of Coimbra, 3000-548 Coimbra, Portugal; Department of Neurology, Coimbra University Hospital, 3000-076 Coimbra, Portugal; Coimbra Institute for Biomedical Imaging and Translational Research (CIBIT), Institute for Nuclear Sciences Applied to Health (ICNAS), University of Coimbra, 3000-548 Coimbra, Portugal; Clinical Academic Centre of Coimbra (CACC), Faculty of Medicine (FMUC), University of Coimbra, 3000-548 Coimbra, Portugal

**Keywords:** PET, quantification, (R)-[^11^C]PK11195, Alzheimer’s disease, textural markers

## Abstract

Alzheimer’s disease is the most common form of dementia worldwide, accounting for 60–70% of diagnosed cases. According to the current understanding of molecular pathogenesis, the main hallmarks of this disease are the abnormal accumulation of amyloid plaques and neurofibrillary tangles. Therefore, biomarkers reflecting these underlying biological mechanisms are recognized as valid tools for an early diagnosis of Alzheimer’s disease. Inflammatory mechanisms, such as microglial activation, are known to be involved in Alzheimer’s disease onset and progression. This activated state of the microglia is associated with increased expression of the translocator protein 18 kDa. On that account, PET tracers capable of measuring this signature, such as (R)-[^11^C]PK11195, might be instrumental in assessing the state and evolution of Alzheimer’s disease. This study aims to investigate the potential of Gray Level Co-occurrence Matrix-based textural parameters as an alternative to conventional quantification using kinetic models in (R)-[^11^C]PK11195 PET images. To achieve this goal, kinetic and textural parameters were computed on (R)-[^11^C]PK11195 PET images of 19 patients with an early diagnosis of Alzheimer’s disease and 21 healthy controls and submitted separately to classification using a linear support vector machine. The classifier built using the textural parameters showed no inferior performance compared to the classical kinetic approach, yielding a slightly larger classification accuracy (accuracy of 0.7000, sensitivity of 0.6957, specificity of 0.7059 and balanced accuracy of 0.6967). In conclusion, our results support the notion that textural parameters may be an alternative to conventional quantification using kinetic models in (R)-[^11^C]PK11195 PET images. The proposed quantification method makes it possible to use simpler scanning procedures, which increase patient comfort and convenience. We further speculate that textural parameters may also provide an alternative to kinetic analysis in (R)-[^11^C]PK11195 PET neuroimaging studies involving other neurodegenerative disorders. Finally, we recognize that the potential role of this tracer is not in diagnosis but rather in the assessment and progression of the diffuse and dynamic distribution of inflammatory cell density in this disorder as a promising therapeutic target.

## Teaser

Castelo-Branco *et al.* showed that textural analysis in Alzheimer’s disease may be an alternative to kinetic modelling in quantifying putative neuroinflammatory changes as measured by (R)-[^11^C]PK11195 PET images. This method may be relevant in the assessment of progression of the diffuse distribution of inflammatory cell density in this disorder.

## Graphical Abstract

**Graphical Abstract:**
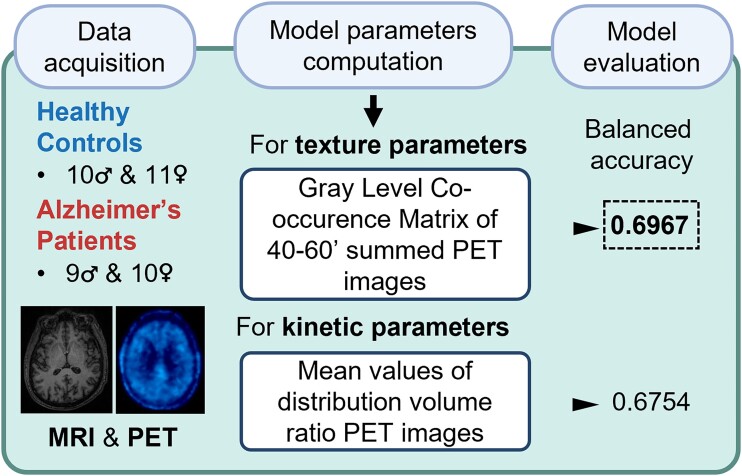

## Introduction

Alzheimer’s disease represents the most common form of dementia worldwide,^1-3^ accounting for an estimated 60–70% of diagnosed cases.^4^ A sizable proportion of the risk of developing Alzheimer’s disease can be attributed to genetic factors.^5^ Increasing age, cerebrovascular diseases, female sex, diabetes, hypertension, obesity and dyslipidemia are other known risk factors for Alzheimer’s disease.^1,5,6^ This condition is considered a slowly progressive disorder^1,7^ as it presents a long asymptomatic phase before the first symptoms appear.^7,8^ The early and most prominent symptoms include progressive memory loss, difficulty with communication, perceptual changes, impaired daily life activities and changes in personality such as increased irritability and anxiety.^1,9^

According to the current understanding of molecular pathogenesis, the main hallmarks of Alzheimer’s disease are the abnormal deposition of amyloid plaques in the brain, composed of β-amyloid peptide (Aβ), and neurofibrillary tangles, containing hyperphosphorylated tau proteins.^2,10,11^ The amyloid cascade theory proposes that Aβ formation is directly responsible for triggering tau phosphorylation and formation of neurofibrillary tangles, leading to neuronal loss and cognitive deficits.^12-14^

For decades, clinical, neurological and neuropsychological examinations were used as the main criteria for diagnosing Alzheimer’s disease.^3,8^ This diagnostic approach changed when technological advances in neuroimaging (MRI and PET) and CSF analysis allowed the development of biomarkers for Alzheimer’s disease.^15^ Because increased amyloid plaque deposition and neurofibrillary tangles can be detected decades before the onset of symptoms, biomarkers reflecting these underlying biological mechanisms are recognized as valid tools for an early diagnosis.^16^ The most relevant ones are CSF biomarkers that directly measure the presence of Aβ and aggregated tau, MRI for visualizing brain atrophy, [^18^F]FDG PET for measurement of brain metabolism and amyloid and tau PET for evaluating the accumulation of amyloid plaque and pathogenic tau, respectively.^7,16,17^ However, imaging biomarkers are limited due to the high cost and access constraints.^17^ For these reasons, neurological exams, cognitive assessments and CSF biomarkers are still the most widely used diagnostic tools for diagnosing Alzheimer’s disease. Recently, a blood test was developed to detect Aβ protein accumulation in the brain.^18^ It promises to predict Aβ levels with >90% sensitivity and specificity compared to PET scanning.^18^ Due to the cost and scalability advantages over current techniques, in the future, these plasma biomarkers may enable broader clinical access and efficient population screening.^9,18^

Inflammatory mechanisms, like microglial activation, are known to be involved in Alzheimer’s disease onset and progression.^2,4,19^ Actually, an amyloid cascade/neuroinflammation theory suggests that Aβ formation activates microglial cells, which release potentially neurotoxic substances, resulting in tau phosphorylation and neurodegenerative changes.^12^ The microglial activated state is associated with increased expression of peripheral benzodiazepine receptors,^20,21^ also known as the translocator protein 18 kDa (TSPO).^22^ On that account, tracers capable of measuring this signature might be instrumental in assessing the state and evolution of Alzheimer’s disease.

Here, we used (R)-[^11^C]PK11195, a radiolabelled specific antagonist of the TSPO that has been established for >30 years in the clinical context of several pathologies.^22^ These include Alzheimer’s disease,^10,13,19-21,23-32^ Parkinson’s disease,^24,33-37^ Lewy body dementia,^38^ Huntington’s disease^39,40^ and amyotrophic lateral sclerosis.^41^ In Alzheimer’s disease, various studies found increased (R)-[^11^C]PK11195 binding in several cortical regions.^13,20,21,24-32^ Two studies found an increased binding in brain regions, such as the parahippocampal, cingulate, middle temporal, superior parietal and superior frontal cortex,^10^ and small clusters in the occipital lobe.^19^ Others were not able to replicate differences in regional (R)-[^11^C]PK11195 binding between controls and Alzheimer’s disease.^31,32^ A longitudinal study found dynamic changes in activated microglial hotspots in six of eight Alzheimer’s patients over 16 months.^27^ Another longitudinal study of the same group supported a similar type of evidence by reporting distinct microglial patterns in different stages of Alzheimer’s disease.^30^ In the mild cognitive impairment group, Fan *et al.*^30^ found decreased levels of (R)-[^11^C]PK11195 uptake over time, whereas in Alzheimer’s disease, a longitudinal increase was observed over 10–18 months. In contrast, Ismail *et al.*^13^ reported that overall levels of inflammation declined over 2 years. These longitudinal results suggested that activated microglia might present distinct dynamic patterns in the evolution of Alzheimer’s disease. A protective anti-inflammatory role may dominate during the acute early phase response followed by a chronic pro-inflammatory response that becomes detrimental,^30,42^ leading to the failure in clearing Aβ plaques.^13^

The methods used to quantify PET neuroimaging data are often based on kinetic modelling of tracers using regions of interest (ROIs).^43-46^ Besides the need for dynamic scanning, since no brain region is devoid of TSPO, an arterial input function is also ideally required to quantify TSPO PET images using kinetic models.^46,47^ Because it involves blood sampling from an arterial catheter, this approach is more invasive and experimentally demanding, especially in frail patients.^47,48^ Therefore, there is a need to have PET quantification methods and metrics based on simpler scanning procedures that provide additional information relevant to the disease and increase patient comfort and convenience.^46^ Apart from these benefits, the diffuse distribution of (R)-[^11^C]PK11195 raises the question of whether alternatives to the traditional ROI-based kinetic approaches should be attempted, which concerns (R)-[^11^C]PK11195.

Gray Level Co-occurrence Matrix (GLCM) textural parameters applied to (R)-[^11^C]PK11195 PET images can provide a statistical description of the spatial characteristics of TSPO.^49^ Accordingly, the present study hypothesizes that whole-brain (grey and white matter) GLCM-based textural parameters may be an alternative to ROI-based kinetic modelling in (R)-[^11^C]PK11195 PET images in Alzheimer’s disease. To achieve this goal, kinetic and GLCM-based textural parameters were computed from (R)-[^11^C]PK11195 PET images of 19 patients with an early diagnosis of Alzheimer’s disease and 21 healthy controls and submitted separately to classification using a linear support vector machine.

## Materials and methods

### Dataset

The dataset used in this cross-sectional study consists of 40 subjects, 19 Alzheimer’s patients and 21 healthy controls matched for age, sex and education. Using the Clinical Dementia Rating (CDR) instrument, we included patients in the same disease stage, with an early diagnosis (<2 years) of probable Alzheimer’s disease at a mild stage of dementia (CDR = 1). All participants performed the acquisitions of the structural MRI and functional (R)-[^11^C]PK11195 PET at the Institute of Nuclear Sciences Applied to Health. The demographic characteristics of the dataset are summarized in Table 1. Alzheimer’s disease participants were assessed according to standard clinical examination procedures from the Memory Clinic of the *Centro Hospitalar e Universitário de Coimbra*. A multidisciplinary team performed all the evaluations and the diagnosis using a consensus approach based on the Diagnostic and Statistical Manual of Mental Disorders fourth edition^50^ and the criteria for probable Alzheimer’s disease dementia of the National Institute of Neurological and Communicative Disorders and Stroke and the Alzheimer’s Disease and Related Disorders Association.^8^ The control group was composed of 21 healthy volunteers from the community without neurologic or psychiatric disorders, with no severe visual or auditory impairment, and eligible for an MRI and PET exam. The inclusion criteria were general cognition within normal ranges and independence in basic and instrumental daily life activities. When required, all participants and their caregivers gave written informed consent for the study conducted according to the Declaration of Helsinki and subsequent revisions. Further, ethical approval was obtained from the ethics committee of the Faculty of Medicine of the University of Coimbra.

**Table 1:** Demographic and clinical characteristics of the study population

	Alzheimer’s disease (*n* = 19) (mean ± SD)	Healthy controls (*n* = 21) (mean ± SD)
Age, years	66.316 ± 7.048	65.857 ± 6.836
Sex, M/F	9/10 (0.900)	10/11 (0.909)
Education	8.895 ± 5.801	11.095 ± 5.513
MMSE	14.611 ± 4.313	—
MoCA	14.368 ± 4.323	24.158 ± 4.413
CDR	1	—

CDR, Clinical Dementia Rating; F, female; M, male; MMSE, Mini-Mental State Examination; MoCA, Montreal Cognitive Assessment; SD, standard deviation. Data are expressed as mean ± SD, except for the M/F ratio (sex).

### Imaging data

#### (R)-[^11^C]PK11195 PET

(R)-[^11^C]PK11195 PET was produced in-house according to published methods.^51^ A Philips Gemini GXL PET/CT scanner (Philips Medical Systems, Best, the Netherlands) was used to perform dynamic 3D PET scans of the entire brain (90 slices, 2 mm slice sampling) and a low-dose brain CT scan for attenuation correction. PET acquisition started immediately after the intravenous bolus injection of a maximum of 370 MBq of (R)-[^11^C]PK11195. Scans were acquired over 60 min (22 frames: 4 × 30 s + 4 × 60 s + 4 × 120 s + 4 × 240 s + 6 × 300 s). PET data were reconstructed using a LOR-RAMLA algorithm, with attenuation and scatter corrections.

#### Structural MRI

Structural MRI data were collected using a Siemens Magnetom TIM Trio 3 Tesla scanner (Siemens, Munich, Germany) with a phased array 12-channel birdcage head coil. We acquired T_1_-weighted structural MRI data at 1 × 1 × 1 mm^3^ spatial resolution, repetition time 2530 ms, echo time 3.42 ms and flip angle 7°.

### PET image quantification

After image acquisition, 3D Slicer, an open-source software program (https://www.slicer.org/), was used to perform the co-registration between PET and structural MRI images. These images were then transformed into the Montreal Neurological Institute space using SPM12, another open-source software program (https://www.fil.ion.ucl.ac.uk/spm/). At last, all images were visually assessed to fine-tune registration when necessary.

#### Kinetic parameters: ROI-based approach

**Figure 1:**
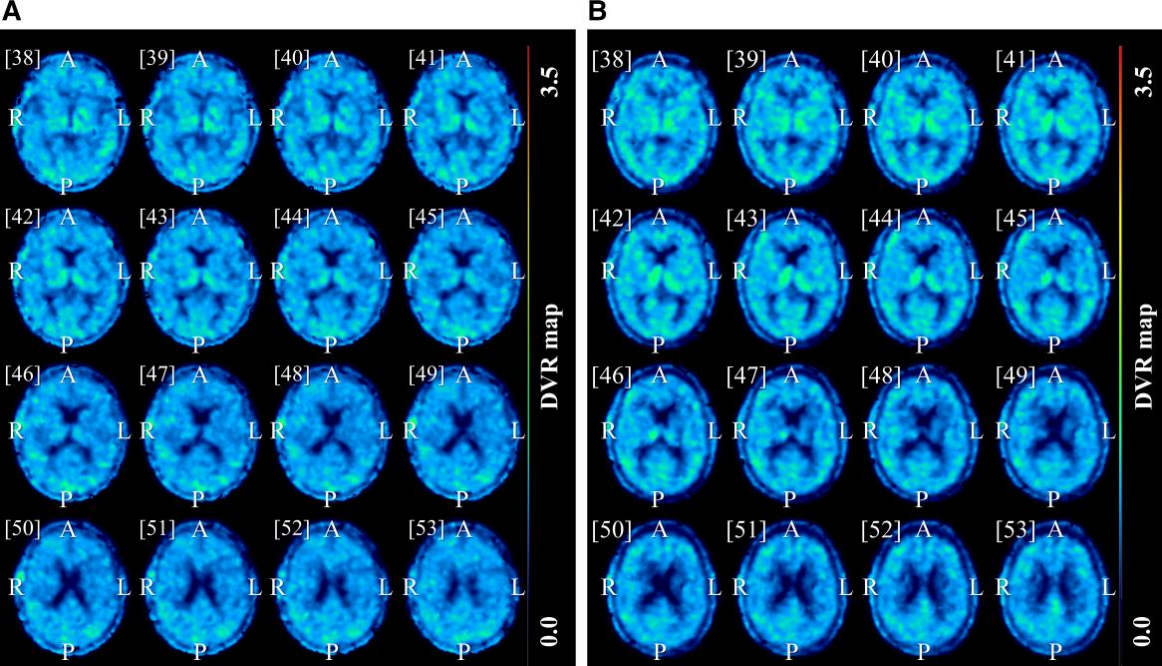
**DVR (R)-[^11^C]PK11195 PET images.** Representative example of a quantitative DVR (R)-[^11^C]PK11195 PET images of an Alzheimer’s disease patient (**A**) and a healthy control (**B**), showing the density of inflammatory cells. This figure was constructed using PMOD software (PMOD, version 4.105; PMOD Technologies, Zurich, Switzerland).

The distribution volume ratio (DVR) was computed at the voxel level for all (R)-[^11^C]PK11195 PET images by applying the Logan^52^ plot method using an in-house made software program implemented in previous works of our institute.^10,53^ The reference region was determined by the supervised cluster analysis algorithm based on four kinetic classes: grey matter without specific binding, white matter, blood and grey matter with specific binding.^54^ A representative example of a quantitative DVR (R)-[^11^C]PK11195 PET image of an Alzheimer’s patient compared to a healthy control is displayed in Fig. 1.

Kinetic parameters were then computed from DVR (R)-[^11^C]PK11195 PET images in Mango free and open-source software (https://ric.uthscsa.edu/mango/) using an ROI-based approach. For each ROI, the mean values were calculated from DVR (R)-[^11^C]PK11195 PET images in the Montreal Neurological Institute space using masks of the anatomical brain region atlas.

#### Texture parameters: whole-brain-based approach

Firstly, (R)-[^11^C]PK11195 PET images were summed at the voxel level from 40 to 60 min post-injection. After this step, the whole brain (grey and white matter) was segmented from all structural MRI images in the Montreal Neurological Institute space using the Extract Brain (BET) Plugin of the Mango free and open-source software (https://ric.uthscsa.edu/mango/). GLCM-based textural parameters were then calculated in MATLAB (MATLAB and Statistics Toolbox Release 2019b, The MathWorks, Inc., Natick, MA, USA) from the summed (R)-[^11^C]PK11195 PET images using these whole-brain masks.

### Feature selection

To minimize overfitting and due to the relatively small sample size (*n* = 40) of the dataset, a 10:1 per-class sample-to-feature ratio was used as a criterion to create robust classifiers.^55,56^ Thus, a subset of four features was selected using the R free software environment (https://www.r-project.org/) from kinetic and GLCM-based textural parameter datasets. Firstly, we computed the correlation between features to avoid cases where correlation between two features was >0.9. After that, we rank the resulting features according to their importance based on a logistic regression model. The four features of major importance for the logistic regression model were selected.

### Classification

The four features selected from kinetic and GLCM-based textural parameter datasets were used separately to build the individual classifiers. These individual classifiers were computed using a linear support vector machine in MATLAB (MATLAB and Statistics Toolbox Release 2019b, The MathWorks, Inc., Natick, MA, USA). We choose to use a linear support vector machine due to its simplicity, wide acceptance and proven good ability for many common classification problems using multivariate medical data.^53^ The label was defined by the clinical Alzheimer’s diagnosis described in the Materials and Methods section, Dataset section. Finally, we use the leave-one-out cross-validation technique to estimate the performance of the individual classifiers. Leave-one-out cross-validation is a particular case of cross-validation that uses the data of a subject to be classified as a single-item test set while the remaining subjects’ data are used to train the classifier.^53,57^ This procedure is repeated until all subjects’ data have been classified once. Then, based on the results of the successive classification tests, the objective measures of test performance and balanced accuracy are calculated.

### Statistical analysis

After assessing for data normality using the Shapiro–Wilk test, the *t*-test or its non-parametric version, the Mann–Whitney test, were used for between-group comparisons of kinetic and textural parameters. These tests were performed using IBM SPSS Statistics for Windows, version 25 (IBM Corp., Armonk, NY, USA).

To assess whether the proportion of Alzheimer’s disease classification is the same between classifiers, the non-parametric Cochran *Q* test was computed in MATLAB (MATLAB and Statistics Toolbox Release 2019b, The MathWorks, Inc., Natick, MA, USA). This test is considered a particular case of the non-parametric Friedman test, used to detect differences in two or more matched sets where the response is binary.^58^

## Results

Tables 2 and 3 show mean ± SD regional and whole-brain values of DVR (R)-[^11^C]PK11195 PET images and GLCM-based summed (R)-[^11^C]PK11195 PET images, respectively. These tables also detail the between-group comparison results of kinetic and textural parameters.

**Table 2:** Mean ± SD regional values of DVR (R)-[^11^C]PK11195 PET images

ROI	Healthy controls (mean ± SD)	Alzheimer’s disease (mean ± SD)	Sig. (two-tailed) (*P*-value^a^)
Frontal lobe	0.895 ± 0.038	0.914 ± 0.041	0.127
Temporal lobe	0.938 ± 0.045	0.933 ± 0.042	0.765
Frontal–temporal space	0.930 ± 0.075	0.924 ± 0.060	0.762
Superior frontal gyrus	0.904 ± 0.038	0.935 ± 0.046	0.027^a^
Middle frontal gyrus	0.915 ± 0.048	0.932 ± 0.047	0.251
Precentral gyrus	0.910 ± 0.038	0.932 ± 0.047	0.251
Inferior frontal gyrus	0.938 ± 0.050	0.944 ± 0.039	0.720
Superior temporal gyrus	0.935 ± 0.047	0.914 ± 0.046	0.149
Middle temporal gyrus	0.977 ± 0.046	0.977 ± 0.045	0.994
Inferior temporal gyrus	0.981 ± 0.056	1.005 ± 0.048	0.155
Amygdala	1.087 ± 0.078	1.084 ± 0.072	0.899
Hippocampus	0.995 ± 0.071	1.017 ± 0.070	0.326
Inferior parietal lobule	0.912 ± 0.044	0.924 ± 0.040	0.360
Superior parietal lobule	0.920 ± 0.055	0.942 ± 0.064	0.249
Precuneus	0.948 ± 0.048	0.969 ± 0.049	0.172

ROI, region of interest; SD, standard deviation. ^a^Significant parametric statistical tests performed between Alzheimer’s and healthy control groups.

**Table 3:** Mean ± SD whole-brain GLCM-based textural parameters of summed (R)-[^11^C]PK11195 PET images

GLCM-based textural parameters	Healthy controls (mean ± SD)	Alzheimer’s disease (mean ± SD)	Sig. (two-tailed) (*P*-value^a^)
Energy	0.621 ± 0.007	0.615 ± 0.007	0.006^a^
Contrast	0.451 ± 0.008	0.459 ± 0.014	0.101
Correlation	0.923 ± 0.024	0.940 ± 0.016	0.021^a^
Variance	24.478 ± 0.933	24.732 ± 1.004	0.208
Homogeneity	0.991 ± 0.002	0.991 ± 2.79E-04	0.180
Sum average	7.243 ± 0.207	7.299 ± 0.224	0.208
Sum variance	88.655 ± 3.507	89.575 ± 3.694	0.239
Sum entropy	0.582 ± 0.009	0.590 ± 0.011	0.025^b^
Entropy	0.850 ± 0.016	0.860 ± 0.017	0.044^a^
Difference variance	0.450 ± 0.008	0.458 ± 0.013	0.081
Difference entropy	0.054 ± 0.012	0.052 ± 0.001	0.133
Information measure of Correlation I	−0.853 ± 0.030	−0.870 ± 0.015	0.021^a^
Information measure of Correlation II	0.803 ± 0.019	0.818 ± 0.016	0.013^a^
Maximal correlation coefficient	0.923 ± 0.024	0.940 ± 0.016	0.021^a^

GLCM, Gray Level Co-occurrence Matrix; SD, standard deviation. Significant ^a^non-parametric and ^b^parametric statistical tests performed between Alzheimer’s and healthy controls groups.

### Kinetic parameters

**Figure 2:**
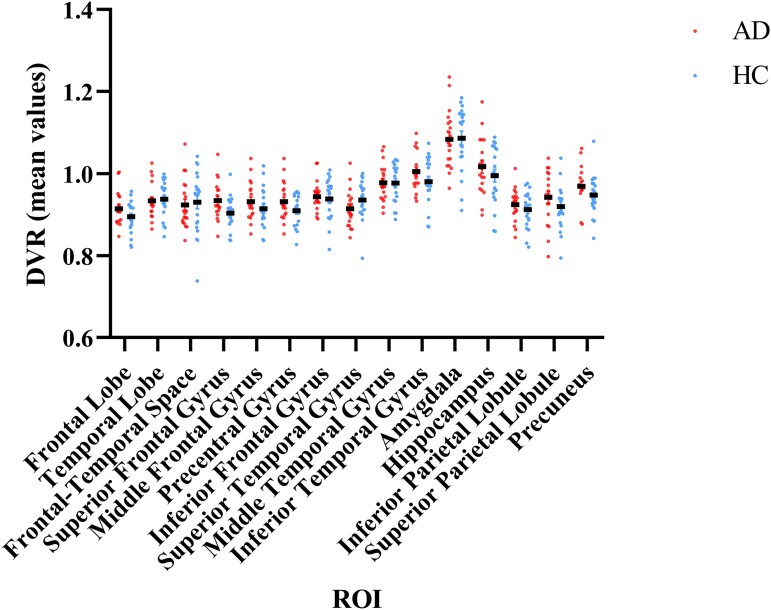
**Scatter plot showing the variance in the binding signal of DVR (R)-[^11^C]PK11195 PET images in the different subject groups.** DVR (R)-[^11^C]PK11195 PET images in healthy control subjects (blue dots) and Alzheimer’s disease (red dots). AD, Alzheimer’s disease; DVR, distribution volume ratio; HC, healthy controls; ROI, region of interest. This figure was constructed using Prism 8 (GraphPad Software, San Diego, CA, USA).

*t*-tests identified significant between-group differences only regarding the superior frontal gyrus [*t*-value (degrees of freedom = 38) = 2294, *P*-value = 0.027]. Overall regional differences of DVR (R)-[^11^C]PK11195 PET images were similar across groups. See Table 2 for details. This result is also illustrated in the scatter plot presented in Fig. 2, showing the variance in the binding signal of DVR (R)-[^11^C]PK11195 PET images in the different subject groups.

### Textural parameters

Significant differences were found between groups regarding the energy, correlation, sum entropy, entropy, information measure of Correlations I and II and maximal correlation coefficient. See Table 3 for details.

### Alzheimer’s disease classifiers

The results of the objective measures of test performance and balanced accuracy for the kinetic and textural-based classifiers and of Cochran’s *Q* test are presented in Table 4. The textural-based classifier showed no inferior performance compared to the classical kinetic approach, yielding a slightly larger classification accuracy (accuracy of 0.7000, sensitivity of 0.6957, specificity of 0.7059 and balanced accuracy of 0.6967) (bold values of Table 4). Using Cochran’s *Q* test, we found no difference between kinetic and textural-based classifiers in Alzheimer’s disease classification [*χ*^2^ statistic *Q* (degrees of freedom = 1) = 0.6923, *P*-value = 0.4054].

**Table 4:** Measures of test performance (accuracy, sensitivity and specificity) and balanced accuracy for the kinetic and textural-based classifiers and Cochran’s *Q* test results

	Kinetic	GLCM-based textural	Cochran’s *Q* test			
**Accuracy**	0.6750	**0**.**7000**	H0: The proportions of (response = ‘AD’) in all groups are equal.			
**Sensitivity**	0.7000	**0**.**6957**	H1: The proportion of (response = ‘AD’) in at least one group is different.			
**Specificity**	0.6500	**0**.**7059**	*χ* ^2^ statistic *Q*	*P*-value	DF	Reject H0 at *α* = 0.05?
**Balanced accuracy**	0.6754	**0**.**6967**	0.6923	0.4054	1	No

AD, Alzheimer’s disease; DF, degrees of freedom; H0, null hypothesis; H1, alternative hypothesis; *Q*, the test statistic value. Cochran’s *Q* test was performed on the hypothesis that the number of classifiers has the same number of successes and failures.

## Discussion

This study aimed to investigate the potential of GLCM-based textural parameters as an alternative to kinetic modelling in quantifying (R)-[^11^C]PK11195 PET images of individuals with Alzheimer’s disease. The focus was to study the role of (R)-[^11^C]PK11195 PET textural parameters as indirect measures of neuroinflammation and disease burden in Alzheimer’s disease. Regarding (R)-[^11^C]PK11195 PET quantification, previous studies have focused on comparing different kinetic modelling techniques using arterial plasma and reference tissue as input functions.^20,47,54,59^ Although the arterial input function is the gold standard methodology, it involves blood sampling from an arterial catheter, which makes this quantification challenging, invasive and an obstacle to a wide application.^47,48,60^ Because no brain region is devoid of TSPO,^47,48^ there is no true reference region for (R)-[^11^C]PK11195,^47^ which also makes the quantification using reference tissue as input function challenging in PET neuroimaging studies using this tracer. Studies have used cluster analysis to extract a reference tissue devoid of TSPO.^19,47,48,54,59,61^ The cluster analysis segments reference tissue voxels based on differences in time–activity curves that are assumed to be without specific binding.^48,54^ Nevertheless, no voxel is actually entirely devoid of TSPO, as this protein is heterogeneously distributed in the brain.^48^ As such, we decided to investigate a different (R)-[^11^C]PK11195 quantification approach that does not require the use of blood sampling or a reference region.

Textural parameters have scarcely been investigated in neurological PET.^47,49^ Mahler *et al.*^62^ found that the shape and texture of the TSPO signal differentiated >96% of multiple sclerosis lesions. Regarding Alzheimer’s disease, texture analysis applied to amyloid PET imaging found that texture or shape features classify Alzheimer’s patients with at least as good accuracy as the classical kinetic modelling approach.^49,63^ These promising sparse findings leave a window of opportunity to implement textural parameters in this field, particularly in TSPO PET.

Using the conventional kinetic ROI-based analysis, we found significant between-group differences just in the superior frontal gyrus (see Table 2 for details), which supports the reported restricted (R)-[^11^C]PK11195 differential binding in fewer anatomical brain regions in Alzheimer’s disease.^10,19^ On the other hand, when using textural whole-brain-based analysis, significant differences were found between groups regarding the energy, correlation, sum entropy, entropy, information measure of Correlations I and II and maximal correlation coefficient (see Table 3 for details). Corroborating these results, the classifier built using the GLCM-based textural parameters yields a slightly larger classification accuracy than the classical kinetic approach (see Table 4 for details). The overall identical performance of both classifiers and Cochran’s *Q* test result (see Table 4 for details) support the stated hypothesis that GLCM-based textural parameters may be an alternative to kinetic modelling in the quantification of (R)-[^11^C]PK11195 PET images of Alzheimer’s disease patients. From these results, we can conclude that (R)-[^11^C]PK11195 PET textural analysis yields similar quantitative markers of inflammatory cell density to kinetic analysis. This textural approach eliminates the need for dynamic acquisition and blood sampling, which increases patient comfort and convenience.

Regarding other neurodegenerative disorders, (R)-[^11^C]PK11195 binding was found elevated in cortical regions of Huntington’s disease patients,^39,40^ in Lewy body participants with mild disease when compared to those with moderate/severe impairment,^38^ in cortical and subcortical structures of amyotrophic lateral sclerosis participants, with variable patterns at the individual level,^41^ and in Parkinson’s disease in cortical regions,^24,35-37^ in the pons and basal ganglia^35^ and in the midbrain at early stages of this disease.^34^ Interestingly, one study found that Parkinson’s disease individuals had comparable or lower regional (R)-[^11^C]PK11195 binding relative to controls.^33^ These results suggest that in other neurodegenerative disorders, inflammatory cell density measured by (R)-[^11^C]PK11195 PET presents a diffuse and dynamic distribution pattern as it seems to happen in Alzheimer’s disease. Thus, we speculate that GLCM-based textural parameters may also be an alternative or complement to kinetic analysis in (R)-[^11^C]PK11195 PET images of other neurodegenerative disorders.

Our study presents a few limitations such as the lack of longitudinal assessment. To overcome this caveat, we used the CDR instrument to ensure a dataset of patients in the same disease stage. CDR can discern very mild impairments, but its weaknesses include the amount of time it takes to implement, subjectivity and relative inability to capture changes over time. Therefore, this work only addresses a relatively early diagnosis (<2 years) of probable Alzheimer’s disease. Due to the progression of chronic inflammation, if we had included patients in advanced stages of Alzheimer’s disease, we would expect to obtain even more detectable changes based on textural analysis. Another limitation of our study is the use of a first-generation TSPO PET tracer, (R)-[^11^C]PK11195, which has been reported to suffer from high non-specific binding and a low signal-to-noise ratio.^59,64,65^

### Additional considerations

Firstly, recent results indicate that TSPO PET more directly reflects the density of inflammatory cells.^66^ Therefore, we reported our results according to (R)-[^11^C]PK11195 as being a measure of microglial activation via the density of its inflammatory cells. Secondly, although the goal of this study was not to find an alternative to other imaging biomarkers in distinguishing Alzheimer’s disease from healthy controls or the relationship between (R)-[^11^C]PK11195 and amyloid deposition, it is still relevant to consider some aspects in this regard.

In a previous work of our group, Oliveira *et al.*^53^ reported that kinetic modelling of [^11^C]Pittsburgh Compound B PET images yielded an accuracy of 96% in Alzheimer’s disease discrimination (accuracy 96%, sensitivity 96% and specificity 95%). Our classifier built using (R)-[^11^C]PK11195 PET images and a similar approach—linear support vector machine and leave-one-out cross-validation technique—shows a lower ability to discriminate Alzheimer’s disease. We recognize that kinetic modelling approaches applied to [^11^C]Pittsburgh Compound B, a PET ligand for *in vivo* evaluation of one of the main hallmarks of Alzheimer’s disease—abnormal deposition of amyloid plaques—are better for discriminating Alzheimer’s disease. Nonetheless, microglial activation is known to be involved in Alzheimer’s disease onset and progression^2,4,19^ and may reflect a dynamic pathological process in this disorder with distinct phases of anti- and pro-inflammatory immune activation.^30,42^ Since (R)-[^11^C]PK11195 represents an indirect measure of this inflammatory mechanism, the main potential of this tracer may be in the assessment and progression of the diffuse and dynamic distribution of inflammatory cell density in this disorder as a promising therapeutic target. Understanding the chronic inflammation pattern mediated by microglia^67,68^ requires the use of other approaches to assess its spatial characteristics. That further justifies our proposed alternative to quantify (R)-[^11^C]PK11195 PET images.

Regarding the correlation between (R)-[^11^C]PK11195 and amyloid deposition, Jorge *et al.*^10^ in a previous work of our group found no significant correlation between (R)-[^11^C]PK11195 and amyloid retention in Alzheimer’s disease, corroborating the results of other studies.^12,29,32^ Conversely, Parbo *et al.*^31^ found clusters with a positive correlation. Others reported positive correlations at both baseline and follow-up.^13,30^ Interestingly, one study showed a significant negative correlation in the posterior cingulate cortex.^28^ These results may indicate that Aβ accumulation is not the primary cause of inflammatory cell density and that these physiological phenomena should be investigated independently.

## Conclusion

In conclusion, our results have shown that GLCM-based textural parameters may be an alternative to conventional ROI-based kinetic modelling in quantifying (R)-[^11^C]PK11195 PET images. The proposed quantification method makes it possible to use simpler scanning procedures, which increase patient comfort and convenience. We further speculate that GLCM-based textural parameters may also be an alternative or complement to kinetic analysis in PET neuroimaging studies involving other neurodegenerative disorders. Finally, we recognize that the role of this tracer is not in diagnosis but may be in the assessment and progression of the diffuse and dynamic distribution of inflammatory cell density in this disorder as a promising therapeutic target.

## Data Availability

The data supporting the findings of this study are available from the corresponding author, M.C.-B., upon reasonable request.

## Abbreviations

Aβ =: β-amyloid peptide
CDR =: Clinical Dementia Rating
DVR =: distribution volume ratio
GLCM =: Gray Level Co-occurrence Matrix
ROI =: region of interest
TSPO =: translocator protein 18 kDa.
